# *Bacillus bombysepticus* α-Toxin Binding to G Protein-Coupled Receptor Kinase 2 Regulates cAMP/PKA Signaling Pathway to Induce Host Death

**DOI:** 10.1371/journal.ppat.1005527

**Published:** 2016-03-29

**Authors:** Ping Lin, Tingcai Cheng, Sanyuan Ma, Junping Gao, Shengkai Jin, Liang Jiang, Qingyou Xia

**Affiliations:** State Key Laboratory of Silkworm Genome Biology, Southwest University, Chongqing, P. R. China; Massachusetts General Hospital Research Institute, Harvard Medical School, UNITED STATES

## Abstract

Bacterial pathogens and their toxins target host receptors, leading to aberrant behavior or host death by changing signaling events through subversion of host intracellular cAMP level. This is an efficient and widespread mechanism of microbial pathogenesis. Previous studies describe toxins that increase cAMP in host cells, resulting in death through G protein-coupled receptor (GPCR) signaling pathways by influencing adenylyl cyclase or G protein activity. G protein-coupled receptor kinase 2 (GRK2) has a central role in regulation of GPCR desensitization. However, little information is available about the pathogenic mechanisms of toxins associated with GRK2. Here, we reported a new bacterial toxin-*Bacillus bombysepticus* (*Bb*) α-toxin that was lethal to host. We showed that *Bb* α-toxin interacted with BmGRK2. The data demonstrated that *Bb* α-toxin directly bound to BmGRK2 to promote death by affecting GPCR signaling pathways. This mechanism involved stimulation of G_αs_, increase level of cAMP and activation of protein kinase A (PKA). Activated cAMP/PKA signal transduction altered downstream effectors that affected homeostasis and fundamental biological processes, disturbing the structural and functional integrity of cells, resulting in death. Preventing cAMP/PKA signaling transduction by inhibitions (NF449 or H-89) substantially reduced the pathogenicity of *Bb* α-toxin. The discovery of a toxin-induced host death specifically linked to GRK2 mediated signaling pathway suggested a new model for bacterial toxin action. Characterization of host genes whose expression and function are regulated by *Bb* α-toxin and GRK2 will offer a deeper understanding of the pathogenesis of infectious diseases caused by pathogens that elevate cAMP.

## Author Summary

Interference with regulation of host signaling by pathogens can alter gene expression, leading to functional disarray in the host cells that causes abnormal division or death. Here, we propose a previously undescribed model for how bacterial toxins subvert host processes via interaction with GRK2 that influences cAMP/PKA signaling. Our findings provide new fundamental information about how bacterial pathogens regulate host signal transduction to cause death, which offers additional perspectives in host-pathogen systems. These findings will help to advance our understanding of bacteria pathogenic mechanism. Furthermore, these might extend to other microbial pathogenesis and assist in designing new or safer strategies against pathogens.

## Introduction

Infectious diseases caused by pathogens result in deaths. Mechanisms of how infection of hosts leads to death have been studied in detail for many pathogens that involve destruction of the cell membrane, inhibition of protein synthesis, or activation of second messenger pathways. However, many questions of bacterial pathogenesis are relatively unexplored, such as which pathogen proteins interact with the host and which infection mechanisms and pathways are commonly triggered by pathogens. These aspects of host-pathogen systems determine the fate of pathogen infections and disease outcomes.

Interactions between host and pathogen in host-pathogen systems are vital for initiating infection. These interactions are associated with regulated pathways that govern a variety of cellular activities and bring about structural and functional disarray within cells that affect survival and fate of the host. An important aspect of host-pathogen systems is the mechanism by which toxins secreted by many pathogenic organisms alter signaling events through interaction with host cell receptors to elevate cellular cAMP concentrations, leading to aberrant activity or cell death. Toxins secreted by *Bordetella pertussis* enhance receptor-mediated GTP-induced activation of adenylate cyclase (AC), resulting in increased cAMP [1]. Cholera toxin increases cAMP level through ADP ribosylation activity toward heterotrimeric G protein [2]. The acylpeptide of *Bacillus subtilis* shows the capacity of inhibitor for cAMP-degrading phosphodiesterases (PDEs) to increase the cAMP levels [3]. The binding of Cry1Ab toxin to Bt-R1 activates G protein to elevate cellular cAMP [4]. These toxins interact with different receptors, provoking cell death by altering signaling pathways to increase cAMP and influence downstream effectors. Therefore, subversion of host signal pathways to change cAMP levels via toxin interaction with receptors is an efficient and widespread mechanism of microbial pathogenesis. In the present study, we have identified and characterized a previously undescribed type of molecular mechanism by which bacterial pathogens increase host cAMP concentration.

G protein-coupled receptor kinases (GRK) regulate G protein-coupled receptors (GPCR) that alter signal transducers with a direct or potential impact in cellular proliferation [5]. GRKs share a common structure comprising a well-conserved central catalytic domain and a C-terminal domain of variable length and structure [6]. Although GRKs show a different tissue expression profiles, subcellular localization, and action [7], they mostly localize at the plasma membrane [8]. Among the family of GRKs, GRK2 have an essential physiological role in the control of growth and development by modulation of GPCRs [9]. Apart from the essential physiological function, changes in GRK2 abundance and activity are an important pathophysiological feature of diseases that have been identified in coronary artery disease [10]. Furthermore, GRK2 has been proposed as a multi-functional protein that interacts with a number of receptors, including EGFR [11] and insulin receptor [12], which are involved in the regulation of several cellular functions controlling larval development. However, the potential involvement of GRK2 in infection of hosts by bacterial pathogens has not been addressed. In this study, we report on a molecular mechanism that used by the toxin of the bacterial pathogen *Bacillus bombysepticus* (*Bb*). The toxin binds to GRK2 to promote larval death associated with interfering GPCR signaling pathway, which activates G protein, increases cAMP and stimulates protein kinase A (PKA). Activation of the cAMP/PKA signaling initiates a series of events that affected homeostasis and fundamental biological processes such as the cytoskeleton or ion channels et al, disturbing the structural and functional integrity of cells, resulting in death. Characterization of host genes whose expression and function are regulated by *Bb* α-toxin and GRK2 will offer a deeper understanding of the pathogenesis of infectious diseases caused by pathogens that elevate cAMP.

## Results

### Effects of *Bb* α-Toxin Protein on Silkworm Lethality

**Fig 1:**
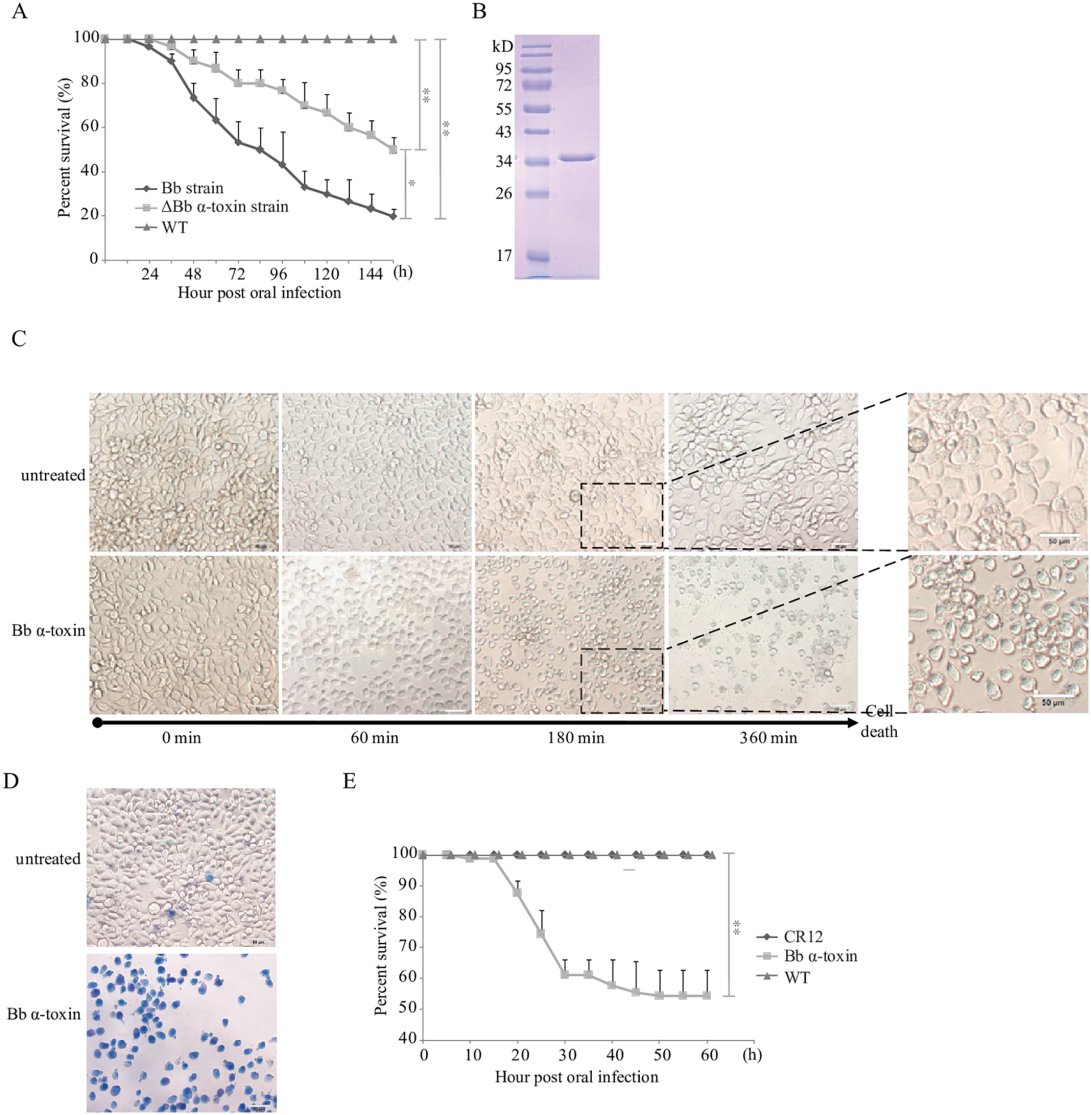
Effects of *Bb* α-toxin protein on the lethality of silkworm. (A) Survival analysis of newly exuviated 5^th^-instar larvae following infection by feeding with *B*. *bombysepticus* wild-type strain (*Bb*), the *Bb* α-toxin deficient strain (Δ*Bb* α-toxin). WT: unchallenged. (B) SDS-PAGE analysis of purified *Bb* α-toxin protein from a prokaryotic expression system. (C) Sequence of cytological changes associated with the progression of *Bb* α-toxin protein (50 μg/mL) as photographed with an Olympus TH4-200 microscope. The long arrows beneath the photographs indicate the relative time for each stage of cell death. (D) Trypan blue staining of nuclei of BmE cells with *Bb* α-toxin protein treatment at 360 min. Blue color represents dead cells; live cells appeared colorless. (E) Survival analysis of newly exuviated 5^th^-instar larvae after *Bb* α-toxin protein treatment for a dose of 50μg per larva. Error bars depict SEM. Statistically significant differences from control samples are indicated; **P<0.01 and * *P*<0.05.

To investigate the molecular mechanisms underlying the regulation of bacteria toxin production by *Bb*, we searched the genome sequence to identify *Bb* toxins responsible for *Bb* pathogenicity. The complete *Bb* sequence was obtained from high-throughput Solexa paired-end sequences [13]. We indentified a predicted extracellular protein that was named *Bb* α-toxin. The deduced amino acid sequence of *Bb* α-toxin displayed limited sequence similarity to a range of pore forming toxins as determined by a BlastP search. To investigate the expression pattern of *Bb* α-toxin during *Bb* pathogens infection silkworm, reverse transcription polymerase chain reaction (RT-PCR) was used. The result shows that *Bb* α-toxin was actually expressed by *Bb* during authentic infection of silkworm (S1 Fig). In order to determine the role of *Bb* α-toxin in the pathogenesis of *Bb*, we generated a *Bb* α-toxin mutant strain (Δ*Bb* α-toxin) by CRISPR/Cas 9 genome editing (S2 Fig) and tested its virulence in silkworm. Interestingly, the Δ*Bb* α-toxin mutant strain demonstrated decreased pathogenicity. Survival analysis of 5^th^-instar silkworm larvae after infection with wild-type *Bb* or Δ*Bb* α-toxin mutant strain showed that only 50% of the larvae infected with Δ*Bb* α-toxin mutant died within 5 days, while 80% of larvae infected with wild-type *Bb* died by this point (Fig 1A). The attenuated virulence of the Δ*Bb* α-toxin mutant strain indicated that *Bb* α-toxin is involved in *Bb* pathogenicity.

To further explore the relationship of biological function between toxic capacity of *Bb* α-toxin and *Bb* pathogenicity, a recombinant protein with an N-terminal His-tag was purified (Fig 1B). In the present works, we examined the morphological changes associated with *Bb* α-toxin protein-treated BmE cells that are silkworm embryo derived cells. As seen in Fig 1C, time-lapse microscopy showed that BmE cells, upon toxin protein exposure, underwent dramatic cytological changes including altered size, shape, and lysis, as compared with untreated viable BmE cells. In order to better characterize the role of *Bb* α-toxin, we used trypan blue to assess cytotoxicity; cells treated with *Bb* α-toxin protein eventually died (Fig 1D). Bioassays showed that larvae died after *Bb* α-toxin protein treatment (S3A Fig), but untreated wild type silkworm (WT) survived (S3B Fig). Another negative control of nontoxic CR12 protein was used to exclude the possibility of effects from protein purification (S3C Fig). Survival rates of newly exuviated 5^th^-instar silkworm larvae were 54.4% for infection *Bb* α-toxin protein, and 100% for CR12 or controls (Fig 1E), with an LD_50_ of 66.1 μg per larva at 5^th^-instar. The survival rate of 4^th^-instar larvae was 32.5% after *Bb* α-toxin infection (Table 1). The approximate LD_50_ value calculated for 4^th^-instar larvae was 34.5 μg per larva. The mortality statistics indicated that *Bb* α-toxin protein was cytotoxic and pathogenic for cells or silkworms.

**Table 1:** Effects of *Bb* α-toxin on exuviated 4^th^-instar silkworm larvae.

	Total no. of larvae treated	Mortality rate	
		No.	%
WT	120	0	0±0
Bb α-toxin	120	81	67.5±13.23**

Mortality rate is total number of insects divided by number that died 5 days after injection of hibitors before *Bb* α-toxin infection.

**P < 0.01.

### Interaction Analysis of *Bb* α-Toxin Protein with G Protein-Coupled Receptor Kinase 2

**Fig 2:**
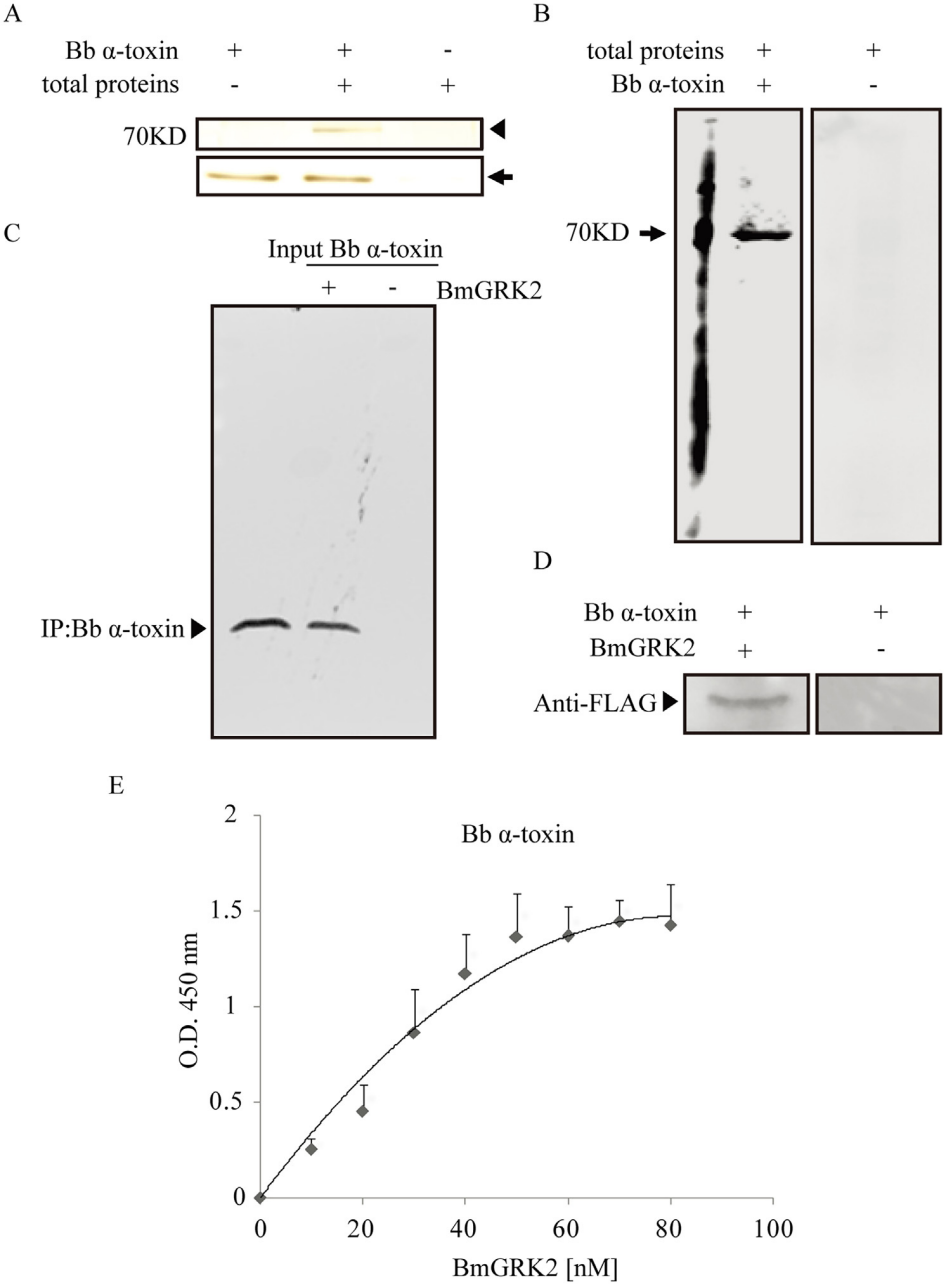
Interaction analysis of *Bb* α-toxin protein with BmGRK2. (A) His-tag pull-down assays of *Bb* α-toxin with total proteins, extracted from midguts BBMV of 5^th^-instar larvae. Lane 1, *Bb* α-toxin protein incubated with PBS as control. Lane 2, *Bb* α-toxin protein incubated with total proteins. Lane 3, NTA beads incubated with total proteins as control to exclude the possibility of any proteins directly interacting with the NTA resin. Arrowhead indicates the pull-down protein of a molecular mass of ~70kDa (lane 2) that bounds with *Bb* α-toxin protein, and arrows indicate the *Bb* α-toxin. (B) Far-western blot of *Bb* α-toxin and total proteins. Total proteins were separated by using 12% (wt/vol) SDS-PAGE and transferred to PVDF membranes. The membranes were either used directly immunoblots using His-tag antibody (lane 1) or used for far-western blot analysis with *Bb* α-toxin protein incubated before adding His-tag antibody (lane 2). Positive bands were observed only at a molecular mass of 70kDa. (C) Co-IP of *Bb* α-toxin and BmGRK2 proteins with Flag tag. Lane 1 shows that *Bb* α-toxin protein was directly immunoblots using the His-tag antibody as a positive control. Lane 2 shows that BmGRK2-Flag protein incubated with the anti-Flag antibody bound to protein G magnetic beads using the BS3 for crosslinking and incubated with the *Bb* α-toxin protein. Lane 3 shows that the anti-Flag antibody bound to protein G magnetic beads incubated with *Bb* α-toxin as negative control. Positive bands were observed only when *Bb* α-toxin/BmGRK2 complexes were present. (D) Far-western blot of BmGRK2 and *Bb* α-toxin proteins. *Bb* α-toxin protein as in *(B)* but with anti-Flag antibody (lane 2) or incubation with BmGRK2 before anti-Flag (lane 1). The arrowhead indicated that BmGRK2 was present. (E) ELISA saturation binding assays of *Bb* α-toxin protein to BmGRK2.

Bacterial toxins kill target cells through receptor-mediation and receptor-disruption of essential cytosolic function. In insects, midgut invasion by pathogens is studied because this is the first line of resistance and immune response. Midgut brush border membrane vesicles (BBMVs) have many receptors for bacterial toxins to participate in pathogenesis [14]. To determine whether or not *Bb* α-toxin protein toxicity involved the midgut, pull-down assays and far-western blots were performed. His-tag recombinant *Bb* α-toxin protein was incubated with total proteins isolated from midgut BBMVs and bound proteins were isolated by affinity chromatography. As shown in Fig 2A, a 70-kDa protein bound to *Bb* α-toxin was detected in pull-down assays. Far-western blots showed that the 70 kDa protein immunologically reacted with His-tag antibody (Fig 2B). The band of 70 kDa was excised from gels for quantitative liquid chromatography-mass spectrometry (qLC-MS) analysis. The LC-MS analysis showed that one of the components in this band is GRK2 (S1 Table), which was originally identified as the kinase that mediates GPCR desensitization and signal transduction [15]. BmGRK2 have transcription activity in BmE cells (S4 Fig).

To confirm the interaction of *Bb* α-toxin protein and BmGRK2, the ORF of *BmGRK2* was cloned by RT-PCR and recombinant BmGRK2-Flag was expressed and purified (S5 Fig). BmGRK2-Flag protein was incubated with anti-Flag antibody bound to beads and crosslinked. After incubation with *Bb* α-toxin protein, BmGRK2-Flag-bound protein was purified by co-IP with anti-Flag antibody. Samples were separated by SDS-PAGE and probed with His-tag antibody (Fig 2C). Anti-Flag antibody bound to beads incubated with *Bb* α-toxin protein was used as a negative control to exclude *Bb* α-toxin directly interacting with beads or anti-Flag antibody. Among the BmGRK2-Flag co-IP products, a product with the molecular mass of *Bb* α-toxin protein was detected by His-tag antibody (Fig 2C). Far-western blots of *Bb* α-toxin and BmGRK2 proteins showed that BmGRK2 interacted with *Bb* α-toxin (Fig 2D). Calculation of the apparent binding affinities obtained by the saturation ELISA binding assays (Fig 2E) revealed that *Bb* α-toxin protein bound BmGRK2 with high binding affinity (*K*_*d*_ = 96.58). The results of these assays confirmed that *Bb* α-toxin protein bound to BmGRK2.

### Correlation of Pathogenicity and Elevated cAMP Stimulated by *Bb* α-Toxin Protein

In the model of GPCR signaling pathway, GRKs terminate GPCR signaling via desensitization of GPCRs by phosphorylation to prevent receptor and G protein association and degrade cAMP [16,17]. cAMP has been implicated in modulation of signaling related to cell death in bacteria to higher eukaryotes [18–20]. Based on *Bb* α-toxin protein binding to BmGRK2, we hypothesized that a cAMP pathway was affected by *Bb* α-toxin protein effort the action of GRK2, involving stimulation of G protein, cAMP and PKA. The production of cAMP is stimulated by G_α_ and subsequent cAMP binds to PKA to activate catalytic subunits of PKA that, in turn, phosphorylate downstream effector proteins [21,22].

**Fig 3:**
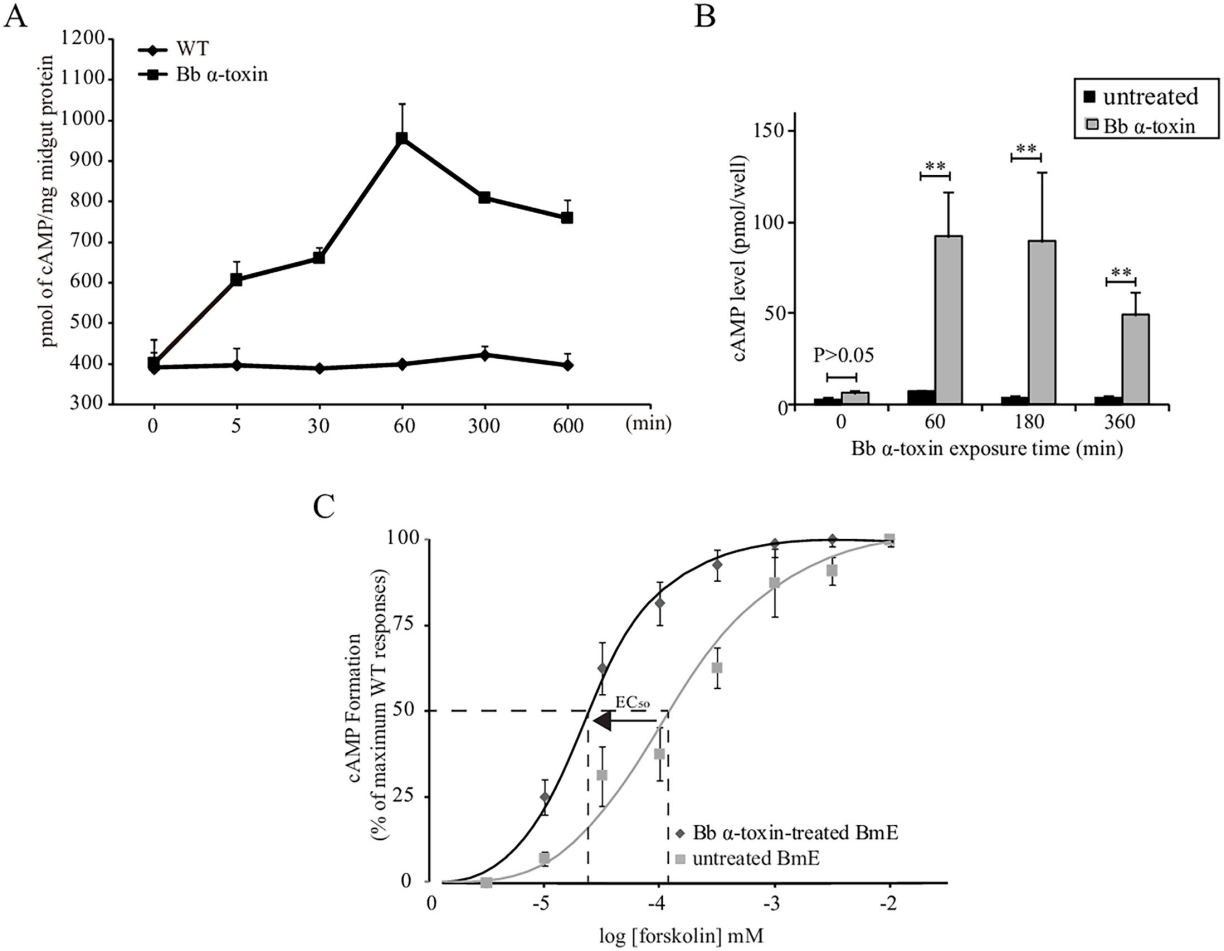
Involvement of cAMP signaling in *Bb* α-toxin protein induced host lethality. (A) Stimulation of midgut intracellular cAMP production by *Bb* α-toxin protein. Larvae were infected with *Bb* α-toxin protein and cAMP was measured. (B) Effect of *Bb* α-toxin protein on cAMP production in BmE cells. cAMP levels were measured in BmE cells after 0, 60, 180, and 360 min of treatment with *Bb* α-toxin protein. (C) Dose response curves for cAMP production in wild-type and *Bb* α-toxin-treated BmE cells in response to treatment with increasing concentrations of forskolin. Data points represent the mean ±SEM of 3 independent replicates.

To test our hypothesis, we analyzed the activity of signaling molecules after *Bb* α-toxin protein oral infection of silkworm larvae. Because elevation of intracellular cAMP levels is hallmark of GPCR pathway activation [23], we measured intracellular cAMP of *Bb* α-toxin protein-treated silkworms and BmE cells at different times. In silkworm larvae continuously exposed to toxin, cAMP production was significantly and consistently increased in the midgut (Fig 3A). In *Bb* α-toxin protein-exposed BmE cells, cAMP production increased in a time-dependent manner (Fig 3B). To determine whether *Bb* α-toxin protein binding to BmGRK2 could exert an effect in GPCR signaling to alter the balance of intracellular cAMP levels, we examined cAMP accumulation in response to forkolin, a direct activator of membrane ACs that is known to promote cAMP production. The EC_50_ for forskolin-stimulated cAMP was significantly reduced in *Bb* α-toxin protein-treated BmE cells as compared with untreated-BmE cells (Fig 3C), indicating that interaction with *Bb* α-toxin protein affected the function of GRK2 in a manner that appeared to lead to sensitization of GPCR receptor signaling. Thus, these results suggested that *Bb* α-toxin protein bound to BmGRK2 to alter the functional action of GRK2 in the GPCR pathway that is critical for stimulating cAMP production.

### Involvement of G_αs_ in *Bb* α-Toxin Protein Pathogenicity

**Fig 4:**
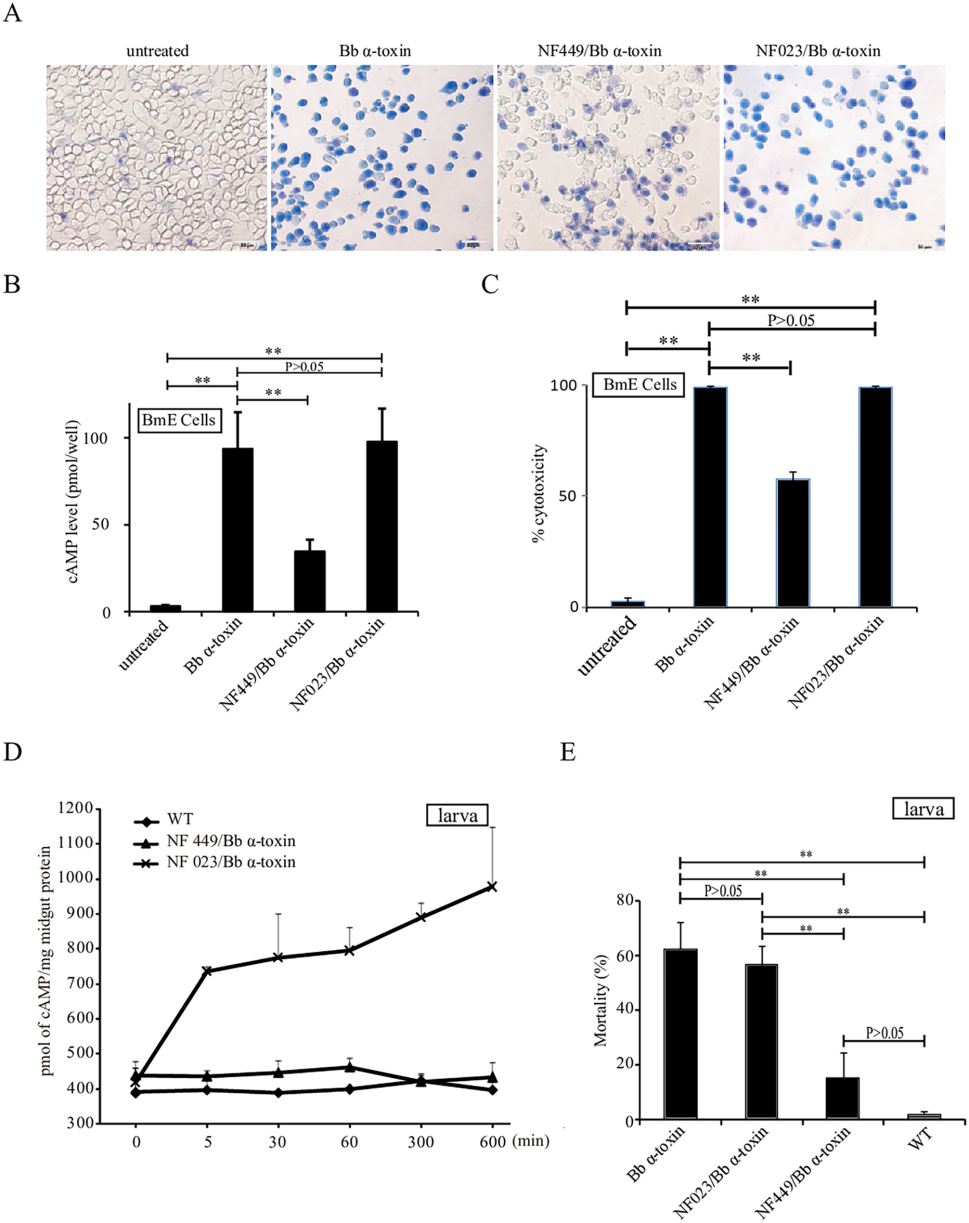
Involvement of Gαs in *Bb* α-toxin protein pathogenicity. (A) Trypan blue staining of nuclei of BmE cells pre-treated with NF449 (1μM) or NF023 (1μM) with *Bb* α-toxin-induced cytotoxicity. NF449-treated cells (NF449/*Bb* α-toxin) were protected from cytotoxic action of *Bb* α-toxin protein whereas NF023-treated cells (NF023/*Bb* α-toxin) were not. Blue color represents dead cells; live cells appeared colorless. (B) Effect of the inhibitors NF449 and NF023 at 1μM on cAMP production in cells. cAMP was measured in BmE cells after treatment with *Bb* α-toxin protein in the presence or absence of the indicated inhibitor. (C) Relative cytotoxicity (%) of *Bb* α-toxin protein in the presence of NF449 or NF023. (D) Assessment of cAMP levels in the presence of NF023 or NF449 under *Bb* α-toxin protein treatment *in vivo*. (E) Effects pathogenicity of *Bb* α-toxin protein in the presence of NF023 or NF449. Mortality rate is total number of 120 insects divided by number that died 5 days after injection of hibitors on *Bb* α-toxin protein treatment. Error bars depict SEM. Statistically significant differences from the control samples are indicated; **P<0.01; *P<0.05.

The production of cAMP is controlled by activation of membrane-bound ACs, which is activated by G_αs_ [21,22]. To ascertain whether G protein activity was involved in the *Bb* α-toxin protein-induced pathway, we used two cell-permeable inhibitors, NF449 [24], that selectively antagonizes G_αs_, and NF023 [25], a G protein antagonist that inhibits the G protein α-subunit G_αi_. BmE cells preincubated (30 min) with NF449 were less sensitive to *Bb* α-toxin protein than cells not treated with inhibitor (Fig 4A). In fact, the concentration of cAMP was significantly reduced in NF449-treated cell when compared to NF449-untreated cells that exposed to *Bb* α-toxin protein (Fig 4B). Moreover, cytotoxicity of *Bb* α-toxin protein decreased by 41.6% when BmE cells were incubated with NF449 (Fig 4C). The *Bb* α-toxin protein-induced BmE cytotoxicity was not significantly inhibited by NF023 and caused cAMP levels to significantly increase as compared to the control (Fig 4A–4C). As can be seen *in vivo*, silkworm larvae that were injected with NF449 and after 30 minutes, treated with *Bb* α-toxin protein had midgut cAMP levels that were less sensitive to *Bb* α-toxin than larvae not treated with NF449 inhibitor and had no effect by NF023 (Fig 4D). To determine whether continuously high cAMP concentrations caused larval death, we also investigated toxicity of *Bb* α-toxin protein with injected inhibitors. The results showed that 62.5% of *Bb* α-toxin protein-treated larvae and 1.67% of controls died (Fig 4E). The toxicity of *Bb* α-toxin protein decreased to 15.0% in larvae injected with NF449 (Fig 4E). The 56.7% lethality for *Bb* α-toxin after injection with NF023 was not different from *Bb* α-toxin protein infection. We concluded from these results that stimulation of G_αs_ protein led to a continuous increase in production of cAMP and was involved directly in the toxicity of *Bb* α-toxin protein, causing death.

### Requirement of PKA Activity in *Bb* α-Toxin Protein Induced Death

**Fig 5:**
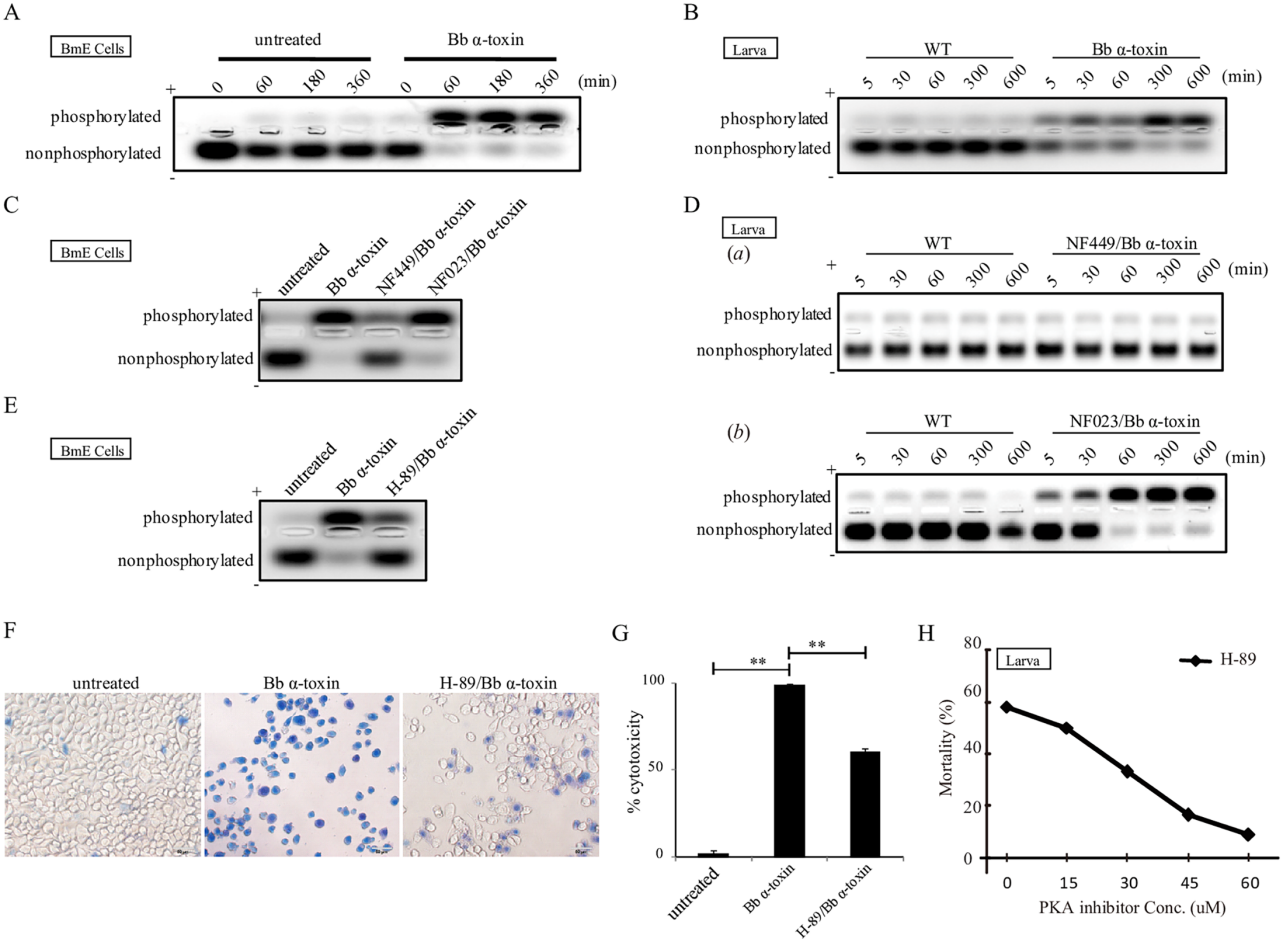
Requirement of PKA activity in *Bb* α-toxin protein induced larval lethality. Representative composite gel of cAMP-dependent phosphorylation of Kemptide; The far right lane represents PKA activity. (A) PKA activity was measured after *Bb* α-toxin protein treatment in BmE cells. (B) PKA activity of larvae following *Bb* α-toxin protein treatment *in vivo*. (C) Effect of PKA activity in the presence of NF449 (1μM) or NF023 (1μM) before the addition of *Bb* α-toxin protein at 50 μg/mL in BmE cell *in vitro*. (D) Assessment of PKA activity in the presence of NF0449 or NF023 with *Bb* α-toxin on the midgut *in vivo*. (E) Effect of PKA activity in the presence of cell-permeable inhibitors of PKA (H-89: 20 μM) with *Bb* α-toxin protein treatment. (F) Trypan blue staining of nuclei of BmE cells pre-treated with H-89 at 20 μM on *Bb* α-toxin cytotoxicity. Inhibitor-treated cells (H-89/*Bb* α-toxin) were protected from the toxic action of *Bb* α-toxin protein. Blue color represents dead cells; live cells appear colorless. (G) Relative cytotoxicity (%) of *Bb* α-toxin protein in the presence of H-89 inhibitor. (H) Percent relative mortality after *Bb* α-toxin with PKA inhibitor (0–60μM) for 30min at specified concentration before addition of *Bb* α-toxin protein.

Generally, PKA activity depends on cAMP concentration [26]. To determine whether the toxicity of *Bb* α-toxin protein was mediated by a cAMP/PKA signaling event, we tested the effects of PKA activity. A representative gel demonstrating the separation of phosphorylated and non-phosphorylated kemptide for PKA effective activity is shown. Qualitative assessment of PKA activity revealed that there were high levels in *Bb* α-toxin protein-treated BmE cells as compared to controls (Fig 5A) or silkworm larvae (Fig 5B); a rough estimate of PKA activity was calculated based on the phosphorylated kemptide. We found that activation of the PKA were terminated by NF449 inhibitor *in vitro* (Fig 5C) and *in vivo* (Fig 5D *a*) after *Bb* α-toxin protein treatment, whereas NF023 had no effect (Fig 5C and 5D *b*). These results indicated that *Bb* α-toxin affected PKA activity. Next, we tested the effects of a PKA inhibitor, H-89; this competitive inhibitor interferes with the utilization of ATP by PKA. H-89 was introduced to BmE cells in a preincubation step followed by the addition of *Bb* α-toxin protein. The results showed that H-89 treatment of BmE cells reduced PKA activity when cells were exposed to *Bb* α-toxin protein (Fig 5E). Moreover, the characteristic morphological changes were partially prevented and blocked cell death by PKA inhibitor (Fig 5F). As can be seen in Fig 5G, the cytotoxicity of *Bb* α-toxin protein decreased by 38.23% when BmE cells were incubated with H-89, indicating that the reduction or elimination of PKA activity abolishes the action of *Bb* α-toxin protein and prevents the death of cells. Indeed, inhibition of PKA activity by injection of H-89 resulted in a dosage-dependent decrease in the lethality of toxin-exposed larvae (Fig 5H). These results demonstrated that inhibition of PKA abolished *Bb* α-toxin action and cAMP-dependent PKA was critical for *Bb* α-toxin action in mediating downstream death activity. Thus, the death of cell or larvae is stimulated by and requires PKA activity.

### Effects of PKA Substrate That Mimics Its Phosphorylation by *Bb* α-Toxin Protein

**Fig 6:**
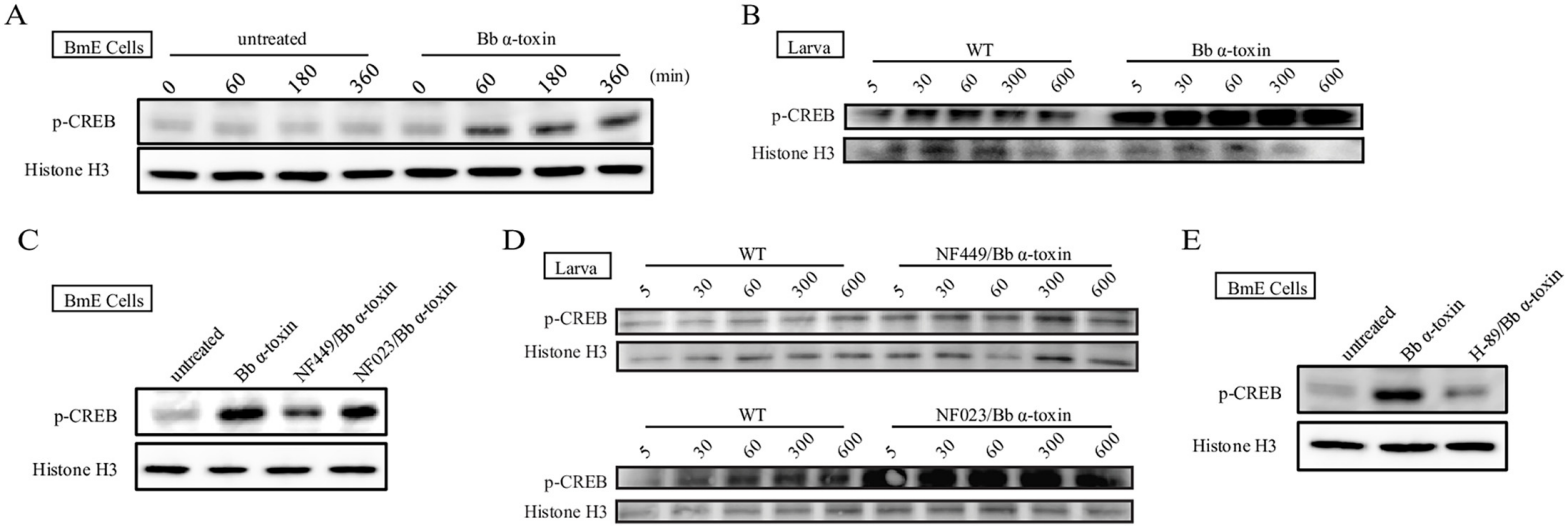
Effects of PKA substrate that mimics its phosphorylation by *Bb* α-toxin protein. (A) Western blot analysis of the expression level of phosphorylated CREB (p-CREB), showing an increase in CREB phosphorylation in after *Bb* α-toxin protein treatment in BmE cells but no such increase in the untreated cells. (B) Immunoblots showing the effect of p-CREB with *Bb* α-toxin protein treatment *in vivo*. (C) *In vitro* assay evaluating the regulatory effect of CREB phosphorylation in the presence of NF449 or NF023 at 1μM with *Bb* α-toxin protein treatment in BmE cells. (D) *In vivo* assessment of CREB phosphorylation in the presence of NF0449 or NF023 with *Bb* α-toxin protein on the larvae. (E) Effect of CREB phosphorylation in the presence of cell-permeable inhibitors of PKA (H-89: 20μM) with *Bb* α-toxin protein treatment. Total proteins were extracted and blotted with antibodies against p-CREB and Histone H3.

Activated PKA phosphorylates substrates that control diverse cellular phenomena. Previous researchers have defined the cAMP-response element binding (CREB) proteins, an inducible transcription factor, as one of PKA substrates that mediate an increase in gene expression in response to cAMP/PKA signaling [27–29]. To evaluate the effect of PKA substrates on the PKA signaling pathway with toxicity of *Bb* α-toxin protein, western blotting analysis was performed to detect the expression of p-CREB, which is an indicator of PKA activity. Phosphorylation of CREB was significantly increased by the addition of *Bb* α-toxin protein in BmE cells (Fig 6A) and larvae (Fig 6B). To examine if the requirement for *Bb* α-toxin protein in CREB-dependent transactivation was restricted to cAMP/PKA-mediated activation, we tested the effects of the inhibitors, NF449, NF023 and H-89. The cAMP level was significantly reduced by NF449 treatment as compared to cells that exposed to *Bb* α-toxin protein (Fig 4B and 4D). NF449 treatment of BmE cells (Fig 6C) or silkworm larvae (Fig 6D) caused a significant decrease in the degree of phosphorylation of CREB, whereas this propensity was absent treated with NF023 (Fig 6C and 6D). Furthermore, pretreatment of BmE cells with H-89 inhibitor significantly decreased the phosphorylation of CREB when cells were exposed to the *Bb* α-toxin protein (Fig 6E). These results were consistent with the transcriptional level of CREB by qRT-PCR analysis (S7 Fig). Therefore, these support the phosphorylation of CREB is occasioned by α-toxin.

## Discussion

In this article, we demonstrated that *Bb* α-toxin protein binding to BmGRK2, a key GPCR regulatory kinase, led to continuous upregulation of its downstream effects, which include stimulation of G_αs_, increase in cAMP and activation of PKA, to induce host death. *Bb* α-toxin protein exerted pathogenicity in toxin treatments of cells and larvae (Fig 1C–1E and Table 1). Overall, our data indicated that the effects of BmGRK2 binding with *Bb* α-toxin lead to a dysregulation in GPCR desensitization that influenced cAMP/PKA signaling, which was associated with toxicity (Figs 2–6). Increased intracellular cAMP levels is a hallmark of activation of cAMP-related signal transduction pathways that mediate cell death or growth [30,31]. Preventing cAMP production by inhibition of G_αs_ with NF449 substantially reduced the pathogenicity of *Bb* α-toxin protein, whereas inhibitor of G_αi_ (NF023) had no effect on toxicity (Fig 4). The physiological effects of cAMP signaling are mediated via PKA effector molecules [32,33]. PKA activity assays showed that continuous, high-level activation of PKA in cell or larvae midguts after *Bb* α-toxin protein treatment was maintained following cAMP increase (Fig 5A–5D). Pretreatment of larvae with H-89, an inhibitor of PKA, protected host from the action of *Bb* α-toxin protein (Fig 5F–5H).

GRK2 regulates GPCR phosphorylation resulting in receptor desensitization that involved in modulation of most physiological processes [5,10]. GRK2 recruitment and activation affects the signals emanating from GPCRs to regulate the balance of intercellular cAMP levels and directly or potentially impacting cell cycle progression and proliferation [34]. Attenuation or depletion of *Drosophila Gprk2* in embryos or adult flies induces dysfunction of muscles, loss of fibers, and flightless behavior [35]. In vertebrates, GRK2 hemizygous mice shows the importance of GRK2 in hypertension [36]. Furthermore, GRK2 ablation causes embryonic lethality, supporting that GRK2 has a central, general role in key cellular processes [37]. Similarly, alteration of GRK2 abundance and activity are associated with inflammation, cardiovascular disease, and tumors, suggesting that alterations contribute to initiation or development of pathologies [10,38,39]. We found that *Bb* α-toxin protein, which was lethal to host, interacted with BmGRK2, indicating that continuous exposure to *Bb* α-toxin had prompted alterations GRK2 and coupled signaling components; in turn, this activated the cell death machinery. Bb α-toxin binding to BmGRK2 has an effect on action of GRK2. This leads to untimely functional interactions of GRK2 with pathways that interrupt GPCR, resulting into impairing cell cycle progression associated with the progression of larvae death. This result reveals a previously unknown activity of GRK2 in silkworm development.

Elevation of intracellular cAMP levels is hallmark of GPCR pathway activation [23]. GRK2 regulates the desensitization of GPCR that leads to rapid degradation of cAMP preventing downstream signal transduction [17,34]. cAMP is ubiquitous and crucial for pathogen-host interactions, which are implicated in modulation of signaling and promoting cell death in a variety of species [18–20]. Induction of cell death through elevation of host organism cAMP is an efficient and powerful evolutionary strategy by which pathogenic microbes overcome a host [1–4,40,41]. We demonstrate that cAMP concentrations consistently increased after *Bb* α-toxin protein treatment. Forskolin-stimulated cAMP is enhanced in *Bb* α-toxin protein-treated BmE cells as compared with untreated cells indicating that GPCR desensitization is reduced. This suggests that the binding of *Bb* α-toxin protein to BmGRK2 reduced the GRK2-mediated desensitization of GPCR signaling that enhanced signaling via cAMP. In mammals, many multipass plasma membrane-bound isoforms of cAMP-producing ACs have been identified [42,43]. Regulation of ACs is primarily by GPCR, which releases a GPCR-associated α-subunit of heterotrimeric G protein (αβγ), that binds to G_α_ with ACs, leading to cAMP synthesis. Depending on the nature of the α-subunit, ACs either are inhibited (αi) or activated (αs) [43]. cAMP production and lethality were prevented by NF449, an inhibitor of G_αs_, after *Bb* α-toxin protein treatment demonstrating that regulators of the G_αs_ protein signaling were critical for cAMP synthesis involvement in the pathogenic toxicity of *Bb* α-toxin protein and increased cAMP levels drives death of host. Unfortunately, the results can not be used to determine how *Bb* α-toxin binding to GRK2 induces GPCR desensitization. However, these problems could be solved if we will identify the target of GRK2 from 90 putative GPCRs [44] and clarity the mechanism of GPCR desensitization in silkworm. Despite its preliminary character, this study can clearly indicate that *Bb* α-toxin protein led to death through *Bb* α-toxin binding to BmGRK2 and altering the balance of intracellular cAMP levels to promote host death.

The cellular effects of cAMP are usually mediated by PKA [26]. Binding of cAMP to PKA activates the catalytic subunits of proteins that phosphorylate a set of target proteins to control diverse cellular phenomena [45]. Disruption of PKA activity results in the destabilization of cell processes [46]. Overexpression of the constitutively active PKA catalytic subunit led to dilated cardiomyopathy and cardiomyocyte hypertrophy [47]. Here, *Bb* α-toxin protein-induced cAMP production was able to trigger the activation of PKA and its downstream effectors, as evidenced by CREB transcription factor phosphorylation. The transcription factor CREB acts downstream of PKA signaling pathways. After phosphorylation on serine, CREB binds to the CREB binding protein (CBP) to regulate the transcription of various target genes involved in cell proliferation, differentiation and survival [48]. Abundant evidence suggest that phosphorylated CREB play a direct role in disease pathogenesis, including mediating the malignant behavior of tumor cells [49] or acute lymphoblastic leukemia [50]. However, it remains to be explored the mechanisms downstream of PKA/CREB signaling participates in the process of disease. Notwithstanding its limitation, this study does demonstrate the essential role of PKA activation in the *Bb* α-toxin protein pathogenic mechanism by experiments showing that mortality changes is impaired if PKA activity is inhibited by H-89. Activated PKA alters downstream effectors to affect cell homeostasis, thereby regulating fundamental biological processes such as blood pressure or metabolism to dismantle cells.

The cAMP/PKA signaling pathway is considered to be involved in metabolism, proliferation and development, and some researchers have further claimed that the stimulation of cAMP/PKA signal transduction pathway represents a novel mechanism for regulating cell death. These can be supported by the injection of cAMP, or ectopic expression of a constitutively activated form of G_α_ or PKA, induced cell death, implicating cAMP as the second messenger in the cell death pathway [51]. Studies with transgenic mouse models have revealed that deregulation of the cAMP/PKA pathway can cause apoptosis [52]. Specifically, as seen in cAMP/PKA pathway mediated cardiomyocyte apoptosis in Grave’s disease, which ca result in heart failure [53]. Deregulation of the cAMP/PKA pathway has been implicated in a range of human diseases [54]. In *Drosophila*, activation of the cAMP/PKA signaling pathway is required for cell death in wing epidermal cells [51]. Further, many pathogens are also known to interfere with host cell signaling to promote cell death via cAMP/PKA pathway [1–5]. The present work demonstrates that in cells and tissues, cAMP/PKA pathway has a major role in the regulation of cell death. Little is known about the downstream molecular mechanisms of cAMP/PKA-triggered cell death. Further analysis of the targets of cAMP/PKA will likely link this signaling pathway with the components that directly regulate cell death.

**Fig 7:**
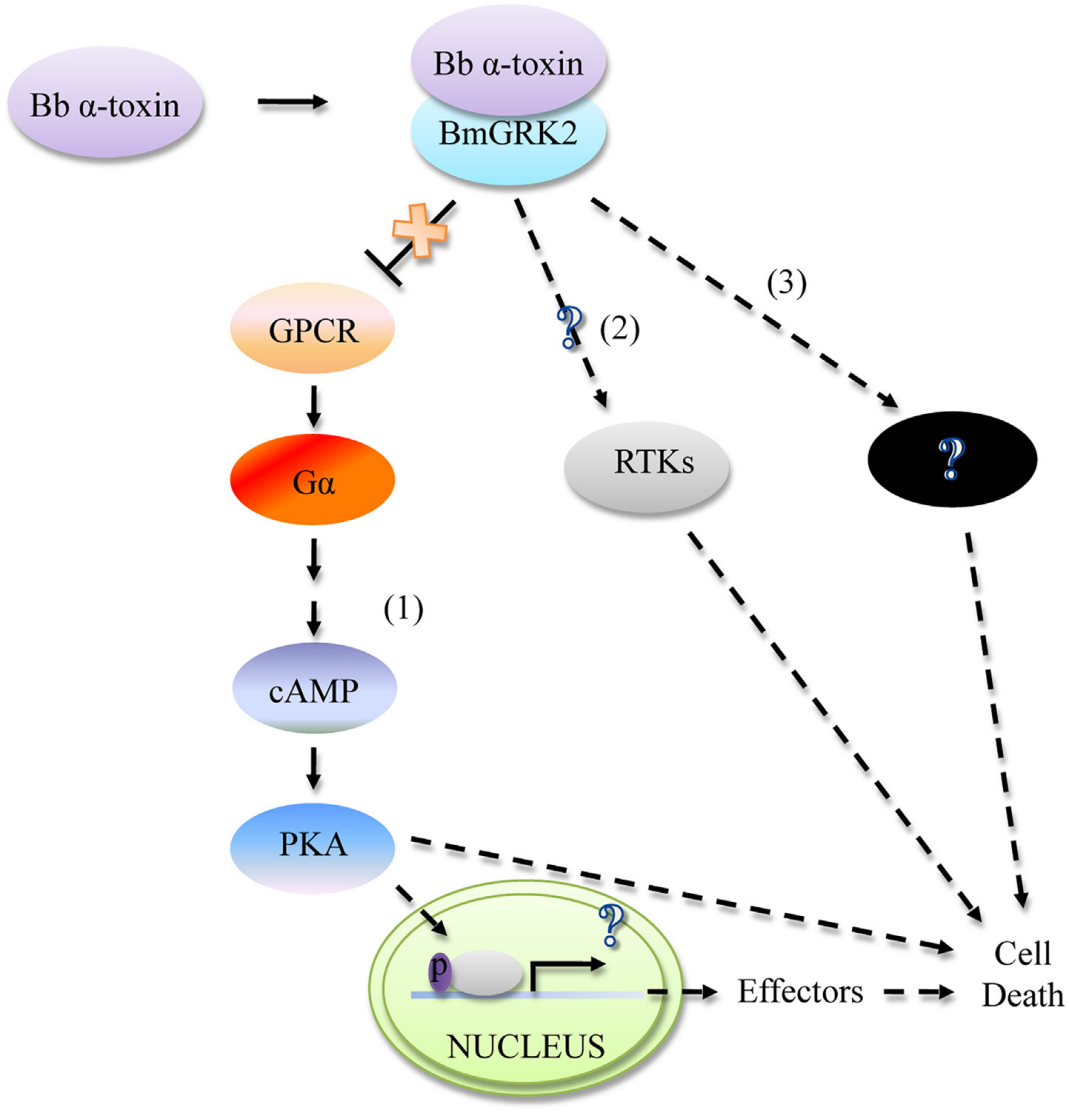
Schematic model for *Bb* α-toxin action. *Bb* α-toxin bound to BmGRK2, a key GPCR regulatory kinase, affecting the modulation of GPCRs signaling pathway, leaded to a continuous up-regulation of downstream activity, which stimulates G_αs_, promotes production of cAMP, and activates PKA. In turn, PKA activation alters effectors that disturb homeostasis and many fundamental biological process that destroy midgut cells, inducing larvae death (pathway 1). Alternatively, *Bb* α-toxin bound to BmGRK2 may also alter the signals downstream of receptor tyrosine kinases (RTKs) that are involved in cell cycle phases, but whether these signaling pathways participate in the *Bb* α-toxin pathogenic mechanism is unknown (pathway 2). Activation of some other signaling pathway is not through pathway 1 or pathway 2 but through other unknown receptors (pathway 3) in *Bb* α-toxin pathogenic mechanism. All of these pathways work together, leading to cell death.

In conclusion, our results demonstrated that host death occasioned by *Bb* α-toxin protein is a complex cellular response to pathology. This paper puts forward a previously undescribed model for bacterial toxin action (Fig 7). The model is a series of events that are confined to or associated with GRK2 action in the pathogenesis of *Bb* α-toxin protein and provide insights into molecular pathogenesis involving G protein activation, cAMP production, and PKA activation. In the model, *Bb* α-toxin binds specifically to BmGRK2, affecting the signal transduction of the GPCR pathway. This action stimulates G_αs_, resulting in accumulation of cAMP and activation PKA (pathway 1). cAMP/PKA signaling is required for cell death [51]. PKA is the key cell death component. Nevertheless, the underlying molecular mechanisms used by cAMP/PKA to control programmed cell death are complex and remain unclear. Determining how the molecular mechanisms of cAMP/PKA signaling pathway leads to death will be useful. Our model suggests a mechanism by which many bacterial toxins challenge hosts through toxin-receptor interaction, manipulating critical reactions associated with cellular responses. Nevertheless, the *Bb* α-toxin protein affects a signaling pathway involving GPCR through GRK2. This mechanism does not resemble classical bacterial toxins that cause larval death through AC activity [1], ADP ribosylation activity that modulates G protein activity [2,55], inhibition of PDEs [3], delivery of cAMP from its own cytosol into cytosol of macrophages [56], or binding to Bt-R1[4] to elevate cellular cAMP concentrations. Likewise, rescue experiments using chemical inhibition show only partial restoration of phenotypes to WT levels. This may suggest additional pathways, or mechanisms, apart from cAMP/PKA that results in Bb a-toxin susceptibility. Alternatively, GRK2 is reported to both positively and negatively regulate signals downstream of receptor tyrosine kinases (RTKs) including IGF-1, PDGF, EGF, insulin, and NGF receptors. These receptors are involved in cell cycle phases [57], but whether these signaling pathways participate in the pathogenic mechanism of *Bb* α-toxin protein is unknown (pathway 2). It is possible that modulation of some other signaling pathway is not through pathway 1 or pathway 2 but through other unknown receptors (pathway 3). Therefore, further characterization of host genes whose expression and function are regulated by *Bb* α-toxin protein will offer a deeper understanding of the pathogenic mechanism of infectious diseases caused by pathogens.

## Materials and Methods

### Experimental Insects and Cell Lines

The silkworm *B*. *mori* strain Dazao (P50) was reared on fresh mulberry leaves at 25°C under a photoperiod of 12 h light and 12 h darkness. Under these conditions, the newly exuviated 4^th^-instar and 5^th^-instar larvae were used to further experiments. *Bb*, a bacterial pathogen of the silkworm, was kindly provided by Professor Yanwen Wang (Silkworm Diseases Laboratory of Shandong Agriculture University, China). The BmE cells [58] were cultured in GRACE medium supplemented with 10% (v/v) fetal bovine serum (FBS) at 27°C.

### Expression Pattern of *Bb* α-Toxin after *Bb* Challenges by RT-PCR

The procedure of *Bb* infection silkworm were according to Lin et al [59]. Total RNA was extracted using Bacterial RNA kit (Omega) following the manufacturer’s instructions. 2 ug of total RNA was then subjected to a Dnase treatment according to the manufacturer’s instructions (Invitrogen). The RNA was reverse transcribed using M_MLV reverse transcriptase according to the manufacturer's instructions (Promega, USA). The cDNA sample was used as the templates for RT-PCR. PCR was done with 27 cycles in a total reaction volume of 25 uL and products were analyzed by electrophoresis in 1% (w/v) agarose gels.

### Design of the Cas9 and gRNA Expression Vector for Screening Δ*Bb* α-Toxin Mutant Stains

To generate the pUC57-gRNA-Cas9 plasmid, we designed that promoter sequence according to Citorik *et al*.[60] and gRNA sequences were placed upstream and downstream of two BbsI enzyme sequences. Cas9 expression was driven by the T7 promoter. The whole sequence was synthesized and inserted into pUC5-T-simple plasmid using Genscript service, forming pUC57-gRNA-Cas9. Guiding sequences of *Bb* α-toxin gRNAs were synthesized as two reverse complement oligos which were annealed and inserted into BbsI-treated pUC57-gRNA-Cas9, forming pUC57-*Bb* α-toxin-gRNA-Cas9. This plasmid was then used to generate the stain Δ*Bb* α-toxin, which contained a deletion of the gene *Bb α-toxin*.

### Construction of Expression Vectors and Recombinant Expression

Genomic DNA of *Bb* was extracted utilizing the E.Z.N.A. bacterial DNA kit (Omega). The ORF of *Bb* α-toxin was cloned into the pET-28a expression vector using the *BamH*I and *Xho*I. Total RNA was extracted from the silkworm midgut tissues using TRIzol reagent (Roche) and reverse transcribed as described [59]. The ORF of BmGRK2 were cloned into the pET-28a expression with Flag tag on the C-terminal ends of the target sequences using *Xba*I and *Xho*I. The correct clones were transformed into *Escherichia coli* BL21 (DE3) to express *Bb* α-toxin and BmGRK2 proteins.

### Cell Treatment and Assay for Cytoxicity

BmE cells were harvested and seeded in 96-well plates (Costar) at a 3×10^4^ cell/well concentration and allowed to grow attached to the bottom surface of the plate. Growth medium was replaced with fresh medium containing *Bb* α-toxin at 50 μg/mL. After addition of *Bb* α-toxin, images of BmE cells were recorded at 0, 60, 180, and 360 min with an Olympus TH4-200 microscope. BmE cells were preincubated for 30 min with NF449 (1μM), NF023 (1μM), or H-89 (20μM), respectively, before the addition of *Bb* α-toxin for cytotoxicity assays. Cell death was determined by typan blue exclusion. These experiments were performed according to the trypan blue staining kit protocol (Beyotime). When observed with a microscope, the dead cells appeared blue and the live cells appeared colorless. The total number of cells per field was counted. Final values represent an average of 6 fields that were randomly selected for each treatment; three separate experiments were performed in triplicate. The BmE cells incubated with *Bb* α-toxin were treated with increasing concentrations of forskolin (0–100 μM) for 60 min and lysed with cAMP Lysis Buffer. In addition, quantitative determination of cAMP, PKA activity, and phosphorylates of CREB with BmE cells were also analyzed after these treatment as below.

### Larvae Treatment and Bioassay of Pathogenic Activity for *Bb* α-Toxin

Silkworm natural infections with *Bb* and Δ*Bb* α-toxin strains were carried out as described previously by Huang *et al*. [61] and all infections were performed with bacterial preparations adjusted to an OD = 100 which correspond to 1.2E11 colony forming units per ml. Virulence assays were performed at least three times. The survival of silkworms after infection with *Bb* α-toxin was investigated. Newly exuviated 4^th^-instar and 5^th^-instar larvae were fed on fresh mulberry leaves coated with purified *Bb* α-toxin for a dose of 50 μg per larva. 120 larvae were tested and recorded for mortality within 5 days. Furthermore, the 5^th^-instar silkworm larvae were injected with NF023 (1 μM/mg), NF449 (1μM/mg), and H-89 (0–60 μM/g) for 30 min, respectively, before *Bb* α-toxin treatment for bioassays. In addition, quantitative determination of cAMP, PKA activity, and phosphorylates of CREB were also analyzed after these treatment as below.

### Protein Isolation, Pull-Down Assays, Far-Western Blots, Co-immunoprecipitation and ELISA Binding Assay

Insect midguts were dissected from silkworm larvae and used to prepare the total proteins of midgut brush border membrane vesicles (BBMVs) by differential precipitation using MgCl_2_ [62] and stored at -80°C until use.

For pull-down experiments, 200 ug total proteins were mixed with 100 ug recombinant *Bb* α-toxin with a His tag and incubated at 4°C with gentle agitation for 12 h. Ni-NTA resin (100 μL) was added and incubated at 4°C for 30 min. After washing five times with PBS, protein was eluted from the resin with eluting buffer (PBS with 500 mM imidazole). Eluted samples were separated on SDS-PAGE and analyzed by qLC-MS/MS which was done by Shanghai Applied Protein Technology Co. Ltd.

For far-western blots, total proteins (50 μg) were separated by 12% (wt/vol) SDS-PAGE and transferred to PVDF membranes. One membrane was used for direct immunoblotting using anti-His antibody (1:8,000) as a negative control. Another membrane was used for far-western blot analysis incubated with *Bb* α-toxin and probed with anti-His antibody (1:8,000) to detect protein interactions. All of the membranes were washed in TBST (20 mM Tris-HCl, pH 8.0, 150 mM NaCl, 0.05 Tween-20) five times, with each wash lasting 10 min, and the procedures as described for far-western blots was performed according to the method of Wu et al. [63].

To identify BmGRK2 protein interacted with *Bb* α-toxin, BmGRK2 with a Flag tag was used as bait in co-IP assays. BmGRK2-Flag protein (100 μg) was incubated with anti-Flag antibody bound to Protein G magnetic beads (Thermo Fisher Scientific) and crosslinked using BS3 (Thermo Fisher Scientific) according to the manufacturer’s protocols. After washing the beads, *Bb* α-toxin (100 μg) was incubated overnight at 4°C with gentle agitation. Complexes were eluted after washing in PBS five times. Absence of BmGRK2-Flag was the negative control. Eluted samples were separated using SDS-PAGE, followed by western blot analysis with anti-His antibody. Far-western blots were performed to identify interactions between BmGRK2 and *Bb* α-toxin as described above. A [add a space] summary of ELISA protocol was described by Lin [64].

### Measurement of cAMP and PKA Activity

After experimental treatments of BmE cells and silkworm larvae as above, samples were harvested and washed in buffered saline solution three times. All of the samples were dissected and sonicated in Cell lysis buffer 5 on ice, and freeze/thaw cycle twice before centrifugation at 600 g for 10 min. Supernatants were used for cAMP assays. cAMP levels were measured using cAMP Assay kits (R&D Systems) according to the manufacturer’s protocols. All samples and standards were assayed in duplicate, and the results were averaged. Concentration were determined by reference to a standard. Statistical analysis was analyzed with GraphPad using student t-tests.

The BmE cells or midgut tissue samples after treatment were harvested, washed in PBS and resuspended in PKA extraction buffer (25 Mm Tris-HCl pH 7.4, 0.5 mM EDTA, 0.5 mM EGTA, 10 Mm beta-mercaptoethanol, 1 μg/mL leupeptin, and 1μg/Ml) that incubated on ice for homogenization. Samples were cleared by centrifugation and Supernatants were used for PKA assay. The qualitative activity measurements of PKA were used PepTag non-radioactive protein kinase assays (Promega, WI, USA) according to the manufacturer’s description.

### qRT-PCR and Western Blot Analysis of CREB Expression

qRT-PCR and western blot was performed as described by Lin [64]. After experimental treatments of BmE cells and silkworm larvae as above, the total proteins were harvested and separated in 12% (wt/vol) SDS-PAGE, and proteins were transferred to PVDF membranes. The p-CREB antibody (1:1,000; Santa Cruz Biotechnology, Santa Cruz, CA) and Histone H3 antibody (1:1000; Beyotime) was used as the primary antibody to detect the phosphorylation of CREB.

### Statistical Analysis

Statistical analysis was analyzed with GraphPad (GraphPad Software, LaJolla, CA) using student t-tests. No significant difference between the session is indicated; P>0.05 and statistically significant differences are indicated; * P<0.05, **P<0.01. The LC50 was calculated using GraphPad Prism software version 5.0 for Windows.

## Supporting Information

S1 Fig Expression profiles of *Bb* α-toxin during *Bb* infection silkworm by RT-PCR. (PDF)

S2 Fig Cas9/gRNA-induced mutations at the *Bb α-toxin* locus in the *Bb* strain. (A) Schematic representation of pUC57-*Bb* α-toxin-gRNA-Cas9 plasmid. (B) RT-PCR analysis of *Bb* α-toxin deficient in Δ*Bb* α-toxin strain. (C) Sequences of mutations. The wild type sequence (WT) is shown at the top and the blue sequence represents the PAM sequence of the gRNA. Within the sequences, deletions are indicated by dashed lines. (D)Sequences of mutations at the targeted *Bb* α-toxin locus by TA-clone sequencing.
(PDF)

S3 Fig Phenotypes of *Bb* α-toxin-treated larvae. Death phenotypes after infection with *Bb* α-toxin (A), larval phenotypes of wild type (WT) (B), and larval phenotypes after treatment with the nontoxic protein CR12 (C).
(PDF)

S4 Fig Expression profiles of BmGRK2 in BmE cell by RT-PCR. The silkworm cytoplasmic actin 3 gene (Bmactin3, GenBank accession no.U49854) was used as the internal control.
(PDF)

S5 Fig Purification of BmGRK2 from Prokaryotic Expression. (PDF)

S6 Fig Larval death phenotype after infection with Bb α-toxin, death phenotype after infection with Bb α-toxin in the presence of NF023 or NF449. (PDF)

S7 Fig Expression profiles of CREB after Bb a-toxin treated BmE cells (A) and larva (B) in the presence or absence of the inhibitors (NF449, NF023 and H-89) by qRT-PCR. (PDF)

S1 Table Identification by QLC-MS/MS of 70kDa protein in pulldown assays. (DOCX)
