# Supplementary figures and images for: Bacillus bombysepticus α-Toxin Binding to G Protein-Coupled Receptor Kinase 2 Regulates cAMP/PKA Signaling Pathway to Induce Host Death
Source: PLoS Pathog. 2016 Mar 29;12(3):e1005527. doi: 10.1371/journal.ppat.1005527 (PMC4811588; doi:10.1371/journal.ppat.1005527)

WT

WT/Bb

5000

3000

2000

1000

750

500

250

100

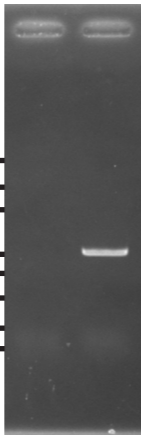

Supplement: S1 Fig — (PDF) [file ppat.1005527.s001.pdf]

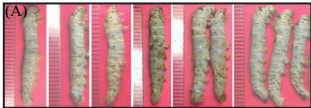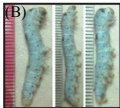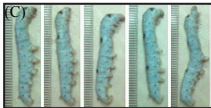

Supplement: S3 Fig — Death phenotypes after infection with Bb α-toxin (A), larval phenotypes of wild type (WT) (B), and larval phenotypes after treatment with the nontoxic protein CR12 (C). (PDF) [file ppat.1005527.s003.pdf]

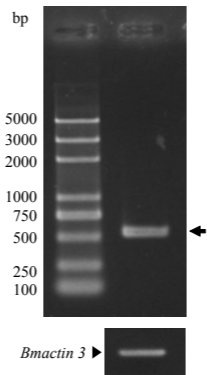

Supplement: S4 Fig — The silkworm cytoplasmic actin 3 gene (Bmactin3, GenBank accession no.U49854) was used as the internal control. (PDF) [file ppat.1005527.s004.pdf]

kDa Marker

170

130

100

70

55

40

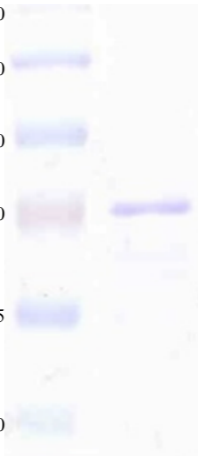

Supplement: S5 Fig — (PDF) [file ppat.1005527.s005.pdf]

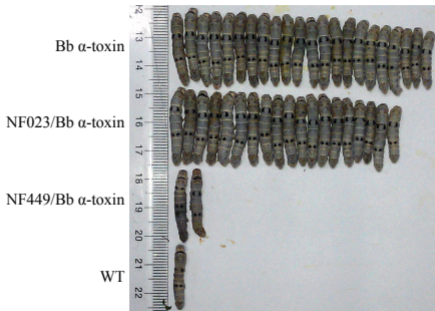

Supplement: S6 Fig — (PDF) [file ppat.1005527.s006.pdf]

A

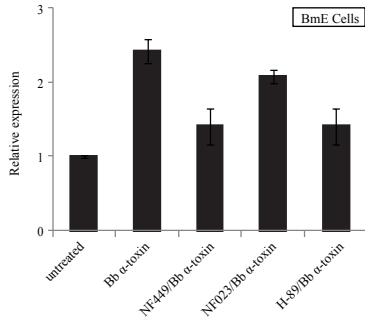

B

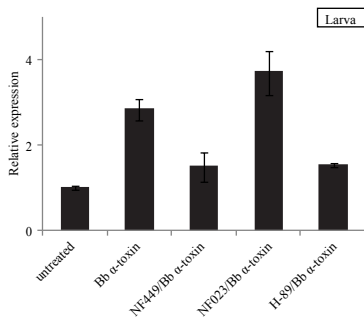

Supplement: S7 Fig — (PDF) [file ppat.1005527.s007.pdf]
