# Supplementary material for: Bacillus bombysepticus α-Toxin Binding to G Protein-Coupled Receptor Kinase 2 Regulates cAMP/PKA Signaling Pathway to Induce Host Death
Source: PLoS Pathog. 2016 Mar 29;12(3):e1005527. doi: 10.1371/journal.ppat.1005527 (PMC4811588; doi:10.1371/journal.ppat.1005527)

A

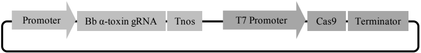

B

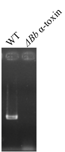

C

Nucleotide sequence:

WT: GCTGATAAACAGATTGCCGTTATTAATACAACTGGTAGTTTTCTAAAAGCAAATCC

ΔBb α-toxin: GCTGATAA--------------------------------------AAGCAAATCC

Amino acid sequence:

WT: FIDDPSADKQIAVINTTGSFLKANPTISDAPIDNYPIPGASATLRY

ΔBb α-toxin: FIDDPSADKSKSYYK*CTY**LPNPWC*CNITLSFTI*CCI*PSR*

D

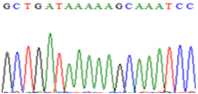

Supplement: S2 Fig — (A) Schematic representation of pUC57-Bb α-toxin-gRNA-Cas9 plasmid. (B) RT-PCR analysis of Bb α-toxin deficient in ΔBb α-toxin strain. (C) Sequences of mutations. The wild type sequence (WT) is shown at the top and the blue sequence represents the PAM sequence of the gRNA. Within the sequences, deletions are indicated by dashed lines. (D)Sequences of mutations at the targeted Bb α-toxin locus by TA-clone sequencing. (PDF) [file ppat.1005527.s002.pdf]
