# Supplementary material for: Bacillus bombysepticus α-Toxin Binding to G Protein-Coupled Receptor Kinase 2 Regulates cAMP/PKA Signaling Pathway to Induce Host Death
Source: PLoS Pathog. 2016 Mar 29;12(3):e1005527. doi: 10.1371/journal.ppat.1005527 (PMC4811588; doi:10.1371/journal.ppat.1005527)
Supplement: S1 Table — (DOCX) [file ppat.1005527.s008.docx]

**Table Supplemental 1**. Identification by QLC-MS/MS of 70kDa protein in pulldown assays.

| Protein IDs | Annotation | MW (kD) |
| --- | --- | --- |
| BGIBMGA001859 | dynein heavy chain family | 477.7 |
| BGIBMGA010415 | Cadherin protein | 199.9 |
| BGIBMGA009149 | carbohydrate binding | 159.0 |
| BGIBMGA010747 | G protein-coupled receptor kinase 2 | 66.6 |
| BGIBMGA009782 | NA | 63.5 |
| BGIBMGA012464 | similar to CG10186-PC, isoform C [*T. castaneum*] | 55.4 |
| BGIBMGA007385 | NA | 25.3 |
| BGIBMGA001749 | NA | 10.4 |

Protein IDs, accession number in SilkDB (http://www.silkdb.org/silkdb/). MW, molecular weight was analyzed by Swiss-Prot/TrEMBL (http://web.expasy.org/compute_pi/).
